# Information exchange between patients with Lynch syndrome and their genetic and non-genetic health professionals: whose responsibility?

**DOI:** 10.1007/s12687-018-0381-5

**Published:** 2018-09-12

**Authors:** Kirsten F. L. Douma, Fonnet E. Bleeker, Niki M. Medendorp, Emmelyn A. J. Croes, Ellen M. A. Smets

**Affiliations:** 1 Department of Medical Psychology, Academic Medical Center/University of Amsterdam, Amsterdam Public Health Research Institute, P.O. Box 22660, 1100 DD Amsterdam, The Netherlands; 2 Department of Clinical Genetics, Academic Medical Center/University of Amsterdam, Amsterdam, The Netherlands; 3 Family Cancer Clinic, The Netherlands Cancer Institute, Amsterdam, The Netherlands; 4 Department Communication and Information Sciences, Tilburg University School of Humanities, Tilburg, The Netherlands

**Keywords:** Referral, hereditary colorectal neoplasms, Genetic testing, Gastroenterology, Communication

## Abstract

Individuals at high risk for Lynch syndrome (LS) should be offered genetic counselling, since preventive options are available. However, uptake of genetic services and follow-up care are currently suboptimal, possibly caused by inadequate exchange of information. Therefore, this qualitative study aims to gain insight in the process of information exchange between patients diagnosed with LS and their non-genetic (i.e., general practitioner, gastroenterologist, gynaecologist) and genetic (i.e., clinical geneticist or genetic counsellor) health professionals concerning referral for genetic counselling and follow-up care. Participants comprised 13 patients diagnosed with LS (8 index patients and 5 of their affected relatives) and 24 health professionals (6 general practitioners, 8 gastroenterologists, 6 gynaecologists and 4 genetic health professionals). Analysis of the interview transcripts was performed in parallel and again after the interviews, following guidelines for qualitative research and using MAXQDA software. The main finding is that patients may ‘get lost’ between health professionals who lack a clear overview of their own and each other’s role and responsibilities in the referral and follow-up care for patients with possible LS. Education of non-genetic health professionals and optimisation of communication between health professionals might help to enable more timely diagnosis of LS and allow patients to address their doubts and questions to the most appropriate healthcare professional.

## Introduction

Lynch syndrome (LS) is an autosomal dominant disorder which gives a 25–70% lifetime risk for colon cancer in most cases (Vereniging Klinische Genetica Nederland 2015). Furthermore, endometrial cancer is generally found in 15–55% of female LS patients, and there is an increased risk for several other types of cancer (Lynch et al. 2009). Since preventive health options (including yearly surveillance) are available, individuals at high risk for LS should be offered genetic counselling, which may reduce morbidity and mortality for colon and endometrial cancer (Lynch et al. 2009).

The chance for a patient to be at risk for LS should be identified by the patient’s general practitioner (GP), gastroenterologist (GE) or gynaecologist, hereafter, together referred to as ‘non-genetic health professionals’ (HPs). To this end, it is essential that they collect enough information on the patient’s family history. However, a survey among hereditary colorectal cancer (CRC) experts showed that (in particular) taking the family history was perceived as poor (Vasen et al. 2010). Non-genetic HPs indeed recognise family history taking (Christianson et al. 2012; Ozanne et al. 2012) and communicating risks (Flynn et al. 2010) as difficult tasks; they also show a lack of knowledge on oncogenetics and express educational needs regarding this item (Klitzman et al. 2012; Prochniak et al. 2012; Sifri et al. 2003). Therefore, identifying patients and relatives at risk and providing them with accurate information and timely referral to a clinical genetic centre may be difficult for them. Not surprisingly, only a small percentage of patients with CRC meeting the criteria for referral for genetic counselling is actually referred (Patel et al. 2016; Singh et al. 2010). Therefore, universal tumour screening has been implemented in the Netherlands for patients aged ≤ 70 years with colon or uterine cancer. However, universal screening does not necessarily lead to higher uptake of genetic services, and clearer roles for the HPs involved are needed (West et al. 2017).

At a clinical genetics centre, a clinical geneticist or genetic counsellor (hereafter, referred to as ‘genetic HPs’) will inform patients about their hereditary risk and the implications of testing and support patients in their decision-making. The diagnostic process benefits from adequate information exchange about cancer in the family and personal cancer history, between the genetic HP, the patient and their non-genetic HPs.

If diagnosed with LS at a genetic centre, patients are advised to undergo regular surveillance and to frequently visit various HPs, particularly GEs and gynaecologists. Unfortunately, GEs may not always follow the screening intervals recommended in the guidelines (Patel et al. 2016). Furthermore, patients can have a significant delay in their surveillance due to, for example, postponing their appointments (Bleiker et al. 2013). To optimise surveillance, better information exchange between patients and their HPs about the relevance and uptake of preventive actions is needed.

It is largely unknown how the different stakeholders perceive their own and each other’s role in the information exchange contributing to suboptimal uptake of genetic services and delay in the follow-up care of patients with LS. Therefore, this study aims to gain insight into how HPs perceive their role in the information exchange between patients and their non-genetic/genetic HPs and what they perceive to be possible causes and solutions for this.

## Methods

### Study procedure and participants

In this qualitative interview study, an introductory letter on behalf of the Netherlands Foundation for the Detection of Hereditary Tumours (NFDHT) was sent to selected index patients of newly registered LS families. The NFDHT is a foundation that holds a nationwide registration of all known LS families in the Netherlands.

Eligible patients were (1) the first in a family to be registered at the NFDHT with the diagnosis of LS (either pre-symptomatic or symptomatic) and (2) registered in the last 3 months (to prevent recall bias). One week after receiving the introductory letter, patients were contacted by the principal researcher (KD) by telephone for additional information. After informed consent, an appointment was made for an interview. A heterogeneous sample of patients with LS that varied in age, gender and disease status (symptomatic vs. pre-symptomatic) was comprised to reflect the most relevant variation in the population.

In-depth, semi-structured interviews were used to generate participants’ views on the information exchange and organisation of care. Interviews were conducted at the patient’s home or by telephone, depending on the patient’s preference. Patients were asked for their consent to approach their (1) non-genetic HPs, i.e., GP, GE and gynaecologist (if applicable), (2) genetic HPs, i.e., clinical geneticist or genetic counsellor and (3) one relative. Relatives were eligible for inclusion when having LS, either symptomatic or pre-symptomatic. The patient was asked to inform their relative about the study by an introductory letter made available by the principle researcher, as privacy regulations precluded directly approaching relatives who had no direct contact with the NFDHT. Relatives willing to participate could send an informed consent form to the researcher. Data of index patients and their relatives are presented together and are referred to as ‘patients’, as they have the same eligibility criteria. The sample size of the patient group was based on data saturation, with a minimum of eight. If consecutive interviews provided no new information, no further patients were approached.

All HPs were sent an introductory letter and were asked to return an informed consent form; if needed, a reminder letter followed after 2 weeks. In case of consent, an appointment was made for a telephone interview. Initially, only HPs involved in the care of participating patients were invited. However, as the response rate among HPs of the interviewed patients was low, HPs of other patients with LS recently registered at the NFDHT were also invited to participate, irrespective of study participation of their patients. Moreover, the gynaecologists initially interviewed knew little about LS making it difficult to acquire a realistic view on the information exchange process; therefore, we approached additional expert gynaecologists involved in the care of patients with LS.

### Data collection

The interview protocols for patients and HPs are presented in Table 1. HPs of participating patients were interviewed about their specific patient and asked to reflect on any differences between this patient and the care for patients with LS in general. The HPs approached directly through the NFDHT were asked to reflect on the care for patients with LS in general.

**Table 1:** Interview schedules

Interview with patients	
A Introduction by researcher	
• Emphasis on voluntary participation	
• Explanation of confidentiality and anonymity	
• Permission for audio taping	
• Short explanation of the goal of the interview	
B Open part	
• Patient’s course of diagnosis of Lynch syndrome	
• Communication about diagnosis	
C Structured part	
Experience with and communication about:	
• Screening (endoscopic and other)	
• Surgery	
• DNA-testing and genetic counselling	
• Risk communication	
Communication with:	
• General practitioner	
• Treating medical specialists	
• Relatives	
D Conclusion of the interview	
• Issues that were not addressed	
• Permission to approach a relative and treating medical specialists and general practitioner	
Interview with medical specialists and general practitioners	
A Introduction by researcher	
• Emphasis on voluntary participation	
• Explanation of confidentiality and anonymity	
• Permission for audio taping	
• Short explanation of the goal of the interview	
B Open part	
• Patient’s course of diagnosis (either about a patient specifically or the course of ‘a typical Lynch syndrome patient’)	
• Communication about diagnosis	
C Structured part	
Experience with and communication about:	
• Screening (depending on the specific expertise)	
• Surgery (depending on the specific expertise)	
• DNA-diagnostics and heredity	
• Risk communication	
• Referral	
Communication with:	
• General practitioner and/or other medical specialist involved	
• Relatives of the patient	
New developments	
Their own role in the care for patients with Lynch syndrome and other hereditary cancers	
D Conclusion of the interview	
• Issues that were not addressed	

Interviews differed slightly between these specialists depending on their level of expertise. Furthermore, some interviews focused on a specific patient whereas others addressed patients in general with Lynch syndrome

Interviews were conducted by the principle researcher (KD) and a research assistant (EC), both with a background in psychology. All interviews were audiotaped and transcribed verbatim. The Medical Ethical Committee of the Academic Medical Centre gave an exemption for formal approval of the study.

### Data analysis

Analysis of the interview transcripts was performed in parallel, and again after the interviews, following guidelines for qualitative research (Braun and Clarke 2006) and using MAXQDA software (MAXQDA, software for qualitative data analysis 1989–2018). Table 2 presents details on the six phases of the analysis.

**Table 2:** Stages of the thematic analysis based on Braun et al. (Braun and Clarke 2006)

Phase	Description of the process
Familiarisation with the data	KD and NM familiarised themselves with the data.
Generating initial codes	KD and NM coded the interviews independently.
Searching for themes	Initial codes were grouped thematically, i.e., working systematically through the entire dataset to identify important aspects that formed the basis of themes, using the interview schedule as guidance.
Reviewing the themes	After each interview, KD and NM compared and discussed codes until consensus was reached. Analysis was inductive, aimed at identifying the most relevant themes.
Defining and naming themes	Gradually, open coding (summarising and categorising the data) was replaced with axial coding (confirmation of codes and identification of broader relationships).
Producing the report	Eventually, common themes were identified. At that time, a researcher (FB) with a background in clinical genetics critically reviewed primary documents and interpretations, as a quality check on the data.

## Results

### Study population

Table 3 provides an overview of the characteristics of the participants. The final sample comprised 13 patients with LS (8 index patients and 5 of their relatives) and 24 HPs (6 GPs, 8 GEs, 6 gynaecologists and 4 genetic HPs). Initially, 14 index patients were asked to participate, of which 8 (57%) consented; reasons for declining were emotional reasons (*n* = 3, e.g., too early after diagnosis, too confrontational), participating in too much research (*n* = 1), no added value perceived (*n* = 1) and unknown (*n* = 1). Through the 8 participating patients, 6 relatives were invited of whom 5 consented to join the study. Details on participation of the HPs are presented in Table 3. Interviews with patients lasted (on average) 60 min and those with HPs 30 min.

**Table 3:** Overview of all participants

Index patient Age (years), gender, symptomatic (symp) versus pre-symptomatic (pre-symp)	Relative gender, relation to patient, symptomatic (symp) versus pre-symptomatic (pre-symp)	General practitioner	Gastroenterologist	Gynaecologist	Clinical geneticist/genetic counsellor
101 62, male, pre-symp	102 male, brother, pre-symp	103	105		
201 57, female, symp, uterine cancer			205		
301 73, female, symp, uterine and colon cancer	302 female, niece, symp, uterine cancer				
401 61, male, pre-symp	402 male, nephew, pre-symp		404		
501 60, female, symp, uterine cancer and colon cancer (2 ×)		503	505	508	
601 37, female, pre-symp	602 female, aunt, symp, uterine cancer				
701 48, male, pre-symp	702 female, mother, symp, uterine and colon cancer		704		
801 49, female, symp uterine cancer					804
		1303	904	1004	1203
		1403	1104	1506	1306
		1503	1605	2305	2206
		1903		2403	
				2603	
Response rates	Six patients gave permission to approach an affected relative; of these, 5 participated and 1 did not respond.	Six patients gave permission to approach their general practitioner of whom 2 participated, 2 declined due to lack of time and 2 gave no reason. Of the 10 general practitioners directly approached, 4 participated.	Six patients gave permission to approach their gastroenterologist, of whom 5 participated and 1 did not respond. Of the 4 gastroenterologists directly approached, 3 participated.	Two patients gave permission to approach their gynaecologist of whom only 1 participated. Of the 11 gynaecologists directly approached, 5 participated.	Five patients gave permission to approach their genetic health professional, of whom 1 participated, 1 was untraceable and 2 declined. Of the 7 genetic health professionals directly approached, 3 participated.

Presented below are results on the process of information exchange related to uptake of genetic counselling, followed by results related to follow-up care. For each of these themes, we present the perspective of patients and those of the various non-genetic and genetic HPs.

### Information exchange related to uptake of genetic counselling

#### Patients’ perspective

About half of the patients explained that coincidence, for example, due to a conversation with a family member or HP, played an important role in discovering that they had LS.

R: I have a sister who is a medical specialist and she has a big network, and when I was diagnosed [with uterine cancer] she came in touch, through her network, with somebody who suggested this might be Lynch. Later, in the hospital, I saw information leaflets about Lynch, and took them home. I had never heard of it, but that was the first time. I thought ‘that could really be about me’. And then, after surgery, I contact a clinical genetic centre myself. 201

Based on the information provided by HPs or relatives, patients often initiated contact with a clinical genetic centre themselves.

Some patients reported that some HPs had done nothing with their relevant family or personal medical history. They felt LS could have been detected earlier if these HPs had actively responded to the information provided.

Overall, the role of the GP as a source of information relevant for referral for genetic counselling was reported to be limited.

In the cases described here, GEs also played a minimal role in the uptake of genetic services. In some patients with symptoms and with a relevant cancer family history, the gynaecologist (rather than the GE) mentioned the possibility of LS. On the other hand, some patients reported that they had not received information about LS from their gynaecologist either. One woman explicitly mentioned that she had the feeling that the gynaecologist did not know much about LS.

Once referred to the clinical genetic centre, most patients perceived the (overall) communication with genetic HPs to be subtle, serious, clear, comforting and careful.

R: Yes, very subtle actually. Of course, in the trajectory before testing he already said something, but we were not really processing it then. You had to hear it [the information], because it is in your family, but you always think you will not have it. So, yes he explained it calmly, did not go into detail about the consequences, but more about the 50% chance for our children. He already said that at that time. And he really took the time for us. 101

However, two patients were less satisfied with the communication by the genetic HP and reported it to be ‘business-like’, i.e., jumping to conclusions and straight to the point.

#### Perspective of general practitioners

Several of the GPs explained that, in contrast to patients with breast cancer, they do not standardly assess the cancer family history in patients with CRC. However, some GPs do this and one had even drawn a family tree.

GPs generally followed the patient’s request to be referred for genetic counselling. GPs reported to generally rely on the cancer family history that patients provide on their own initiative. Frequently, but not always, patients brought information from relatives diagnosed with LS to the consultation. GPs reported to provide very little explanation about LS to their patients at the time of referral, as they lacked the knowledge or did not perceive this to be their responsibility.

I: And when he came for a referral, what did you discuss with him? Did you already discuss Lynch syndrome?

R: I did, to be honest, did not even know the term that became clear to me later. And while looking it up it became clearer to me what it is. But I did not know the term. 103

#### Perspective of gastroenterologists

All GEs said they standardly assess their patients’ family history, although one admitted to do this only in patients with CRC. Often, GEs did not know the precise risk of developing cancer in LS, but did not consider this to be important.

R: It is obviously elevated. But you know, I am not really focusing on the percentages. First of all, you can never remember them and, moreover, it says nothing about the individual patient. 205

GEs who had consulted only a few patients with LS mainly followed the lead of other HPs. In contrast, GEs who consulted many patients with LS felt that HPs at non-university hospitals and GPs lack knowledge and often lack time. Therefore, some of them suggested that patients should be seen by GEs specialised in LS and that care should be centralised in hospitals specialised in LS.

R: However I do notice, that patients who are under surveillance elsewhere, and get genetically tested (and referred to the GE in the academic hospital) indicate that they appreciate that we talk for 45 min to an hour and discuss al ins and outs of hereditary cancer with them. Because they have never done that with their peripheral doctor. 1605

#### Perspective of gynaecologists

Similar to GEs, gynaecologists mentioned that they standardly assess a cancer family history. When gynaecologists are the first to discuss the possibility of LS, they feel that patients can be overwhelmed.

R: Well, what I notice is that often… that for a family it comes as thunder in a clear sky. That nobody has ever thought of it. That I am the first to put the family on this track, that strikes me. 1506

#### Perspective of genetic health professionals

According to genetic HPs, the information available to them at the time of referral is not always sufficient, and they perceived large differences in the information provided by the referring HPs. To get more information on the family history, all genetic HPs send a questionnaire to patients before the first visit.

R: Well there are large differences depending on who is referring. When a referral comes from a GP it is usually rubbish, no information in it [the referral letter] or ‘grandmother had something’, but they do not even know if it was from father’s or mother’s side. If it [the referral letter] comes from a GE it is usually much better. 1203

Genetic HPs felt that most of the non-genetic HPs lack the knowledge to identify patients at risk for LS and to give them the correct information. Some of them suggested centralising care via experts who see a considerable number of patients with LS. They mentioned that they try to inform and educate GPs via copies of their letters.

R: I always send a letter to the patient himself/herself, these are as simple as possible and quite extensive, and a copy goes to the GP and to the referring specialist if that is someone else other than the GP. The effect is that the GP is also educated. 1203

Concerning communication with the patient, most of the information that genetic HPs reported to give to patients is standardised. However, they also report to adapt the information to the foreknowledge and intellectual level of the patient.

I: When a patient comes for a first visit to the clinic, which information do you give to a patient about Lynch syndrome?

R: Eh…well that is quite minimal if it is patients in whom a diagnosis has never been established. In contrast, whenever, patients come in for a pre-symptomatic counselling after the diagnosis of LS has been established in the family, then it is different. But it differs very much. Sometimes they do not know much about it, which is possible when affected relatives are not very closely related, but if they are relatively smart and young and their brother went through the whole process, well then they know what they are talking about. 1306

### Information exchange related to follow-up care for patients with Lynch syndrome

#### Patients’ perspective

Patients were aware that their GP was informed about the genetic test result; however, most patients did not discuss LS and its consequences with their GP. Three patients never talked with their GP about what LS entails and its consequences. Of those who did speak with their GP, which was mostly initiated by the patient, most felt that these GPs were not knowledgeable about LS. For example, one patient took the letter with the genetic test result to his GP. In contrast, another GP had asked about the emotional implications of the genetic test result. Two other patients mentioned how they had extensively discussed with their GP what type of surveillance was possible and needed.

R: So after the diagnosis I have sought contact with my GP and I have given her the information I got from the clinical genetic centre. And I have discussed with her which type of screening I wanted to do and what I have to do. So she cooperated with that straight away, yes. 601

Remarkably, when consulting their GP for other physical complaints, patients felt insecure as to whether the GP took their LS diagnosis into account.

R: I have said to my brothers and sisters ‘will the GP receive an alert, when he types in our names, that a pop-up or so mentions that we have Lynch syndrome?’ 101

Patients did not know whether the GP and other HPs were informed about the results of a colonoscopy and other screening interventions, but assumed so. They reported that, after availability of the DNA test results, they would appreciate more streamlined communication between the HPs involved regarding the policy following a LS diagnosis.

Most patients considered the GE to be their primary caregiver for their LS. Patients felt a need for a dedicated contact person specialised in LS to whom they could address their questions and concerns. However, generally, they felt that the amount of time to address their questions during a normal consultation was too limited.

#### Perspective of general practitioners

As also indicated by the patients, most GPs reported to barely discuss LS with their patients. They felt responsible for referring patients for follow-up care and also for providing support. To be able to properly inform patients, all GPs reported that they had to search for more information on LS. They would like to have rapid access to information and information specifically tailored for GPs.

R: Yes, I think that we need some kind of central point where we can get information very quickly, but we must not be buried under continuing education and lectures and information and magazines that give us more information about a lot. Because we cannot manage that. 103

Several GPs mentioned that they were not regularly informed by GEs about the endoscopic surveillance, while others reported to receive letters or were unsure about whether they were informed by the GE. Some GPs did not know whether patients received surveillance other than endoscopic screening. GPs appreciated the letter from the genetic HP; generally, they only had contact with the GE via letters.

R: I do not get an yearly letter from the gynaecologist or GE or whatever, so I assume everything is alright. 1403

#### Perspective of gastroenterologists

GEs considered it normal practice to discuss LS with their patients. However, they differed with regard to how much they explain to their patients. At a minimum, they felt a need to discuss the elevated risk for CRC and the need for regular surveillance. Most GEs wanted to communicate risks in such a way that it would motivate patients to undergo regular surveillance.

R: Yes. And I always try to focus on the positive…yes, it is very nasty to get such a diagnosis, and it is not nice to undergo such screening, but with this screening you are better protected than someone from the general population that does nothing. So, I find that a relatively positive message. 704

In general, GEs considered it important to inform other HPs about their patients. GEs informed GPs by letter, although not every time a patient came for screening; they were cautious about possible information overload. For specialists in the same hospital, information was available in the electronic health record. However, most GEs did not inform specialists outside their own hospital because they considered this to be too much effort and because of assumed information overload for their colleagues.

R: Yes, and internally of course nowadays we use the computer because that includes all specialists […].

I: Yes, but there is not a standard message going to them.

R: No, that is not necessary. Otherwise you would turn crazy of all those letters. 1605

Some GEs were dissatisfied with the lack of communication with gynaecologists; however, most felt that this complaint was unjustified as they were not providing sufficient information to gynaecologists themselves. GEs who consulted many patients with LS perceived it as their responsibility to educate other HPs and to take the lead in the care for these patients.

R: Yes, we send a letter to the peripheral hospital the patient goes to, in which we explain that they have had an informative consultation including, if necessary, the details that are of importance. And then the follow-up continues in the other hospital. Some peripheral doctors do not mind, but others see this as an infringement on their ego if I may say so. 1605

GEs felt a need to foster a relationship with their patients, but also clearly delimited their own role. They wanted to differentiate between their own involvement and that of the GP in order to prevent patients from visiting them for every physical complaint. They saw an important role for a case manager to streamline questions related to LS.

R: I would like to prevent that people with Lynch syndrome run directly to me when they have physical complaints.

I: that you become kind of a GP?

R: Yes, that happens sometimes and that runs us crazy, that is not possible, we have so many patients, if they all are going to see us as GPs. Of course, when it is colon related than it is alright, but I think the GP should screen whether it is necessary if a specialist has to look at the patient or not. 704

#### Perspective of gynaecologists

Some gynaecologists perceived their role in the care of patients with LS as the regular contact person functioning as a sounding board, others perceived themselves as just a part of the larger picture, and one perceived it as the person taking the control.

Gynaecologists varied in their level of knowledge regarding surveillance for LS. Some did not know the exact advice for preventive surveillance, while others were aware of the guideline and the latest updates.

About half of the gynaecologists thought that patients knew a lot about LS and therefore perceived no need to inform patients, while the other half spent a lot of time explaining LS and checking the knowledge level of the patient; these latter gynaecologists generally had more experience with LS.

Most gynaecologists standardly informed the GP by letter. Gynaecologists informed the specialists within the hospital either through the electronic health record or by a letter. The communication with specialists residing in other hospitals differed; most gynaecologists send a letter to the GE but not to other specialists involved, e.g., the urologist.

Gynaecologists were unsure as to whether they consulted all patients that require their surveillance. They felt it depends on the individual GE whether a patient is referred to them or not. They suggested that a case manager could be helpful in coordinating care for these patients or, as one mentioned, that care should be centralised in a few hospitals. Some feel that for patients, the coordination of surgery could be improved by better information exchange with GEs.

R: Well, what I think is important is that if GEs from elsewhere, for example, diagnose a colon cancer and a patient needs surgery, then sometimes it happens that the uterus remains while I would have wanted it removed in the same surgery, either by myself or the GE. So, that is important, that people know that and that we are updated and are included in each other’s information loop. I think that is an important point for improvement. That every GE that sees a lady with Lynch syndrome asks them if they undergo gynaecological screening and put this gynaecologist in the CC and the other way around. 2603

#### Perspective of genetic health professionals

All genetic HPs send the GP a letter with the DNA test result. Some also standardly called the GP to discuss the DNA test result.

R: I always ask patients, ‘do you appreciate it if I call your GP if I find a new mutation or if you turn out to be a carrier?’ 804

Genetic HPs differed in their opinion on the necessity to be informed about follow-up care by other specialists. From a medical viewpoint, they did not consider this necessary. However, some genetic HPs said that (theoretically) this could lead to differences in surveillance advice or an update of family letters.

R: As long as it makes no difference in the counselling to the family, then for me I do not need that. However, if something is added, like with this woman they find an urothelial carcinoma in the bladder, then I would like to know that. But if everything fits within what we have already discussed, then it does not add anything. 804

## Discussion

To contribute to reducing the risk of LS being undiagnosed, this study explored the perspective of patients with LS and those involved in their care regarding the information process related to the uptake of genetic counselling and subsequent follow-up care. We conclude that patients with LS may ‘get lost’ in the diagnostic process and follow-up track. This implies that a diagnosis might be missed or the referral process may be unnecessarily delayed. Patients experience that some HPs have limited knowledge and perceive the communication between HPs to be suboptimal. Additionally, some do not know who to turn to with questions and concerns at the time of the referral process and during follow-up care.

### Why do patients get lost during the referral process?

From a patient’s perspective, a lack of knowledge was perceived in the non-genetic HPs during the referral process. Overall, HPs could communicate more proactively with each other, e.g., ask other specialists for more information on the cancer history or proactively inform colleagues, which might increase the chance of a timely referral and reduce delay in the diagnosis of LS. Like the patients, genetic HPs observe a lack of relevant knowledge about LS in non-genetic HPs, which could lead to frustration (as indicated in our quote). However, genetic HPs generally expressed a need and a willingness to educate non-genetic HPs. The GPs in this study indeed acknowledged their limited knowledge on LS. As a consequence, they are unaware of, or feel insecure about, routinely and systematically performing a cancer family history assessment. Moreover, GPs might feel hindered to discuss genetics because of their lack of knowledge (Houwink et al. 2011). This is unfortunate because, in the Dutch healthcare system, GPs function as gatekeepers and are in a unique position to assist patients (Houwink et al. 2011). GPs may be reminded of sources of information such as websites about genetics for GPs (e.g., https://www.primarycaregenetics.org, www.huisartsengenetica.nl or http://www.genetics.edu.au/health-professionals/genetics-in-general-practice).

In contrast, GEs and gynaecologists did not perceive themselves to have a limited understanding of LS. They reported to routinely investigate a patient’s family history, albeit without using checklists. As a consequence, they may fail to identify patients at high risk for LS that should be referred (Douma et al. 2016).

Moreover, patients may get lost in the process as a consequence of HPs not having a clear view of their own and each other’s role and responsibilities regarding referral and follow-up care. In this study, GEs perceived a leading role for themselves in referring at-risk patients for genetic counselling. This is in line with the conclusion of West et al. that oncologists and gastrointestinal specialists, together with the pathologist, should play an important role in initiating screening for hereditary CRC (West et al. 2017). Remarkably, this leading role was not always perceived as such by the patients in our sample. This might be due to uterine cancer in four of the eight index patients; in their case, the initiative for referral was taken by their gynaecologist. This illustrates that it also depends on the specific patient as to who is the best person to initiate referral to a genetic service.

### Why do patients get lost during follow-up care?

The main reason for this was the lack of information exchange between HPs. In general, patients assumed that their HPs informed each other about their findings, but they did not know for sure. HPs themselves also feel insecure regarding to what extent they need to inform each other about the follow-up care of their patients. GPs are not sure if they are adequately informed by GEs, while GEs agree that they do not consistently inform other HPs. Professionals are reluctant to inform each other because of the degree of effort this requires, the perceived lack of urgency and fear of causing information overload; the latter has been reported as a barrier to effective communication between the different HPs involved (Hall 2005).

Additionally, most patients mentioned that the diagnosis is hardly discussed with their GP. As a consequence, they feel unsure as to whether the GP is aware of their LS and the possible implications for their overall health. However, some patients discussed screening options or the emotional consequences of LS with their GP. The interviews with GPs confirm this variation in approach.

How does the perspective of patients relate to the role HPs themselves perceive in follow-up care? While GPs and patients agree that they hardly discuss LS with each other, GEs seem to perceive a prominent role for the GP. GEs delimit their own role and prefer to refer patients to the GP. Thus, the roles and responsibilities of the different HPs in the follow-up care of patients diagnosed with LS are unclear to themselves.

What do patients want? During follow-up care, patients have a need for a central person to whom they can ask questions related to LS, as they generally experience HPs to have too little time. Our results suggest that they prefer the GE rather than the GP to take this role as central caregiver. However, GEs seemed ambivalent about fulfilling this role, but consider specialised nurses or case managers. Furthermore, patients would appreciate more streamlined communication between HPs on the procedural policy following a LS diagnosis.

### Implications

Our findings demonstrate the importance of educating both GPs and other HPs about the guidelines, including assessment of a cancer family history, criteria for referral for LS and for follow-up care. Results suggest that GPs are unaware of the information that is available to them on the web and of available continuing educational courses (Houwink et al. 2015).

Our study also suggests that the information exchange between HPs could be organised in clearer pathways which explicitly state the role of all parties involved (West et al. 2017), for example, including structured family history and letters after each contact to all treating physicians in and out their hospital including GPs. Several HPs recommended organising care in specialised centres or expert clinics. This is supported by the observation that families who are seen at high-risk clinics or teaching hospitals are at an advantage in terms of risk recognition (Overbeek et al. 2008; Patel et al. 2016). A case manager or patient navigator (often a nurse practitioner) assigned to at-risk families could help to channel patients’ questions (Paskett et al. 2011; Paskett et al. 2017). Genetic HPs could function as a consultant for their non-genetic HP colleagues in the follow-up care of the patients.

### Strengths and limitations

A strength of this study is that it included the views of a diverse group of patients and HPs involved in their care. By including the whole network around the patient, we obtained a complete view on the information process between all these stakeholders.

This study also has some limitations. First, to increase the number of respondents, we also included medical specialists unrelated to a patient who participated in the study. These specialists provided their general views on the care for LS patients, rather than describing the specifics of a particular case. This might have led to a social desirability bias in their responses.

Second, participating medical specialists may have a relatively higher interest in LS. In contrast, some GPs seemed to be inclined to participate on behalf of their involvement with a patient, rather than because of their interest in LS.

Third, some results may be specific for the Dutch healthcare system. For example, in the Netherlands, until recently only a geneticist ordered genetic testing and, therefore, a thorough family history assessment and adequate referral for genetic counselling by other HPs are essential. This might be less important in countries in which HPs, such as the GEs, are allowed to order genetic tests themselves and where genetic testing is done more routinely.

### Conclusions

We conclude that patients may get lost between HPs that do not have a clear overview of their own and each other’s role and responsibilities in the referral and follow-up care for patients with a possible LS diagnosis. As a result, diagnoses may be missed, the referral process may be delayed, and confusion may arise during follow-up care, both in patients and among professionals. Education of non-genetic HPs and defining clearer pathways in which appointments are made about the information exchange flow between HPs could help to overcome these problems.

Interviews differed slightly between these specialists depending on their level of expertise. Furthermore, some interviews focused on a specific patient, whereas others addressed patients in general with Lynch syndrome.

The numbers refer to the identification numbers we gave each individual interviewed.
